# 1,4-Dimethyl­piperazin-1-ium 3-hy­droxy-2-naphtho­ate

**DOI:** 10.1107/S1600536812005375

**Published:** 2012-02-17

**Authors:** Gemma E. Craig, Carla Johnson, Alan R. Kennedy

**Affiliations:** a Department of Pure and Applied Chemistry, WestCHEM, University of Strathclyde, 295 Cathedral Street, Glasgow G1 1XL, Scotland

## Abstract

The reaction of 1,4-dimethyl­piperazine and 3-hy­droxy-2-naphthoic acid gives the title 1:1 salt, C_6_H_15_N_2_^+^·C_11_H_7_O_3_^−^, with a singly protonated piperazinium cation. In the crystal, a single N—H⋯O hydrogen bond links the cations and anions into discrete pairs and the aromatic anions stack along the crystallographic *a*-axis direction. This results in layers of cations and anions alternating along the crystallographic *c*-axis direction. An intra­molecular O—H⋯O hydrogen bond is also present.

## Related literature

For general descriptions of the salt selection process in the pharmacy industry, see: Stahl & Wermuth (2002 ▶); Gould (1986 ▶); Serajuddin (2007 ▶). For structures of monoprotonated 1,4-dimethyl­piperazinium, see: Clemente *et al.* (1999 ▶); Marzotto *et al.* (2001 ▶). For systematic structural studies of structure–property relationships of salts in a pharmaceutical context, see: Arlin *et al.* (2011 ▶); Kennedy *et al.* (2011 ▶). For the Cambridge Structural Database, see: Allen (2002 ▶). For a related aryl carboxyl­ate structure, see: Burchell *et al.* (2001 ▶).
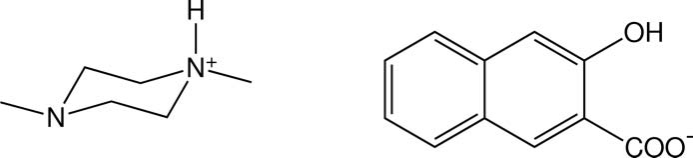


## Experimental

### 

#### Crystal data

C_6_H_15_N_2_^+^·C_11_H_7_O_3_^−^
*M* *_r_* = 302.37
Monoclinic, 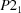

*a* = 5.8772 (16) Å
*b* = 10.892 (2) Å
*c* = 12.562 (2) Å
β = 100.29 (2)°
*V* = 791.2 (3) Å^3^
*Z* = 2
Mo *K*α radiation
μ = 0.09 mm^−1^
*T* = 293 K
0.20 × 0.15 × 0.08 mm

#### Data collection

Oxford Diffraction Xcaliber S diffractometer
7855 measured reflections
2996 independent reflections
1608 reflections with *I* > 2σ(*I*)
*R*_int_ = 0.119

#### Refinement

*R*[*F* ^2^ > 2σ(*F* ^2^)] = 0.046
*wR*(*F* ^2^) = 0.121
*S* = 0.83
2996 reflections
203 parameters
1 restraint
H-atom parameters constrained
Δρ_max_ = 0.14 e Å^−3^
Δρ_min_ = −0.16 e Å^−3^

### 

Data collection: *CrysAlis PRO* (Oxford Diffraction, 2010 ▶); cell refinement: *CrysAlis PRO*; data reduction: *CrysAlis PRO*; program(s) used to solve structure: *SHELXS97* (Sheldrick, 2008 ▶); program(s) used to refine structure: *SHELXL97* (Sheldrick, 2008 ▶); molecular graphics: *ORTEP-3* (Farrugia, 1997 ▶) and *X-SEED* (Barbour, 2001 ▶); software used to prepare material for publication: *SHELXL97*.

## Supplementary Material

Crystal structure: contains datablock(s) global, I. DOI: 10.1107/S1600536812005375/lh5414sup1.cif

Structure factors: contains datablock(s) I. DOI: 10.1107/S1600536812005375/lh5414Isup2.hkl

Supplementary material file. DOI: 10.1107/S1600536812005375/lh5414Isup3.cml

Additional supplementary materials:  crystallographic information; 3D view; checkCIF report

## Figures and Tables

**Table 1:** Hydrogen-bond geometry (Å, °)

*D*—H⋯*A*	*D*—H	H⋯*A*	*D*⋯*A*	*D*—H⋯*A*
O3—H3⋯O2	0.82	1.81	2.541 (3)	148
N2—H2⋯O1^i^	0.91	1.71	2.613 (3)	174

Symmetry code: (i) 
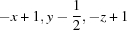
.

**Fig. 1.**
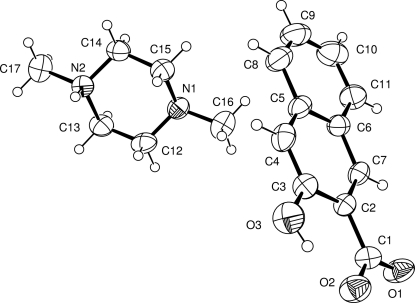
Contents of the asymmetric unit of (I). Displacement ellipsoids are drawn at the 40% probability level.

**Fig. 2.**
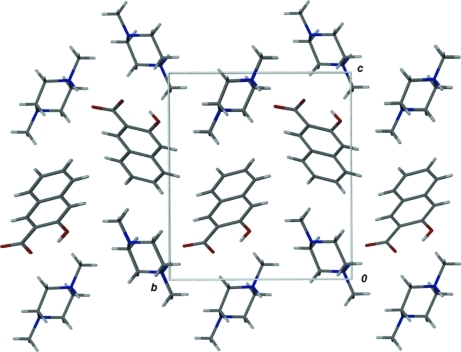
Packing of (I) viewed along the a direction. Note the stacked anions down the a direction and the layers of cations and anions that alternate along the c direction.

## supplementary crystallographic information

### Comment

The influence of solid-state structure on salt selection in the pharmacy industry is of ongoing interest and importance (Stahl & Wermuth, 2002; Gould, 1986; Serajuddin, 2007). As a contribution towards this we have recently investigated series of salt structures based on both protonated tertiary amines and anions derived from aryl carboxylic acids (Arlin *et al.*, 2011; Kennedy *et al.*, 2011). Combining these two themes we present here the structure of 1,4-dimethylpiperazinium 3-hydroxy-2-naphthoate (I).

Reaction of equimolar amounts of 1,4-dimethylpiperazine and 3-hydroxy-2-naphthoic acid in an aqueous environment gave a 1:1 salt with the base monoprotonated to give 1,4-dimethylpiperazinium, see Fig. 1. A search of the Cambridge Structural Database (Allen, 2002) found only three other examples of structures containing the monoprotonated base. All were RMCl_3_ structures (*M* = Co, Cu, Zn) with the non-protonated amine forming a bond to the metal centre (Clemente *et al.*, 1999; Marzotto *et al.*, 2001). In contrast there were 54 hits for the di-protonated cation, [C_6_N_2_H_16_]^2+^, with organic anions. This later group includes all the carboxylate based anions, including the only other aryl-carboxylate reported (Burchell *et al.*, 2001). The molecular geometries of both ions in (I) are unexceptional. The near equal C—O lengths of the carboxylate group confirm its deprotonated nature. The piperazine ring exists in a chair conformation with equatorial methyl groups. Note that the C—N bond lengths of the protonated N2 atom are systematically longer than those involving the neutral atom N1.

Both potential hydrogen bond donors are utilized. The hydroxy group makes an internal hydrogen bond with one O atom of the carboxylate group whilst the the second O atom accepts an intermolecular hydrogen bond from NH of the cation, Table 1. This relatively limited hydrogen bonding results only in discrete cation-anion pairs being formed with no extended hydrogen bonding network. The aromatic anions stack along the crystallographic *a* direction in an offset manner such that the closest contact is between C2 of the carboxylate substituted ring and C11 of the non-substituted ring (C2···C11' = 3.576 (4) Å, ' = *x* + 1, *y*, *z*). The polar orientation of neighbouring anion stacks is reversed along the crystallographic *c* direction and this results in alternating layers of anions and cations as shown in Fig. 2.

### Experimental

Addition of an equimolar amount of 1,4-dimethylpiperazine to an aqueous slurry of 3-hydroxy-2-naphthoic acid with stirring and heating to 323 K gave a clear solution. After cooling to room temperature, colourless crystals of (I) were deposited after 3 days.

### Refinement

All the H-atoms were placed in geometric positions and refined in riding modes. N—H and O—H distances were set to 0.91 and 0.83 Å respectively, with the best fit orientation of the OH group to observed electron density being found by allowing rotation about the C—O bond. C—H distances of 0.93, 0.96 and 0.97 Å were adopted for CH, CH_2_ and CH_3_ groups respectively. *U*_iso_ = 1.2*U*_eq_ of riden atom, except for CH_3_ and OH where *U*_iso_ = 1.5*U*_eq_ of riden atom.

### Crystal data

**Table:**

C_6_H_15_N_2_^+^·C_11_H_7_O_3_^−^	*F*(000) = 324
*M**_r_* = 302.37	*D*_x_ = 1.269 Mg m^−^^3^
Monoclinic, *P*2_1_	Mo *K*α radiation, λ = 0.71073 Å
Hall symbol: P 2yb	Cell parameters from 1466 reflections
*a* = 5.8772 (16) Å	θ = 2.5–31.3°
*b* = 10.892 (2) Å	µ = 0.09 mm^−^^1^
*c* = 12.562 (2) Å	*T* = 293 K
β = 100.29 (2)°	Fragment, colourless
*V* = 791.2 (3) Å^3^	0.20 × 0.15 × 0.08 mm
*Z* = 2	

### Data collection

**Table:**

Oxford Diffraction Xcaliber S diffractometer	1608 reflections with *I* > 2σ(*I*)
Radiation source: fine-focus sealed tube	*R*_int_ = 0.119
Graphite monochromator	θ_max_ = 26.0°, θ_min_ = 2.5°
Detector resolution: 16.0268 pixels mm^-1^	*h* = −7→7
ω scans	*k* = −13→13
7855 measured reflections	*l* = −15→15
2996 independent reflections	

### Refinement

**Table:**

Refinement on *F*^2^	Secondary atom site location: difference Fourier map
Least-squares matrix: full	Hydrogen site location: inferred from neighbouring sites
*R*[*F*^2^ > 2σ(*F*^2^)] = 0.046	H-atom parameters constrained
*wR*(*F*^2^) = 0.121	*w* = 1/[σ^2^(*F*_o_^2^) + (0.0613*P*)^2^] where *P* = (*F*_o_^2^ + 2*F*_c_^2^)/3
*S* = 0.83	(Δ/σ)_max_ < 0.001
2996 reflections	Δρ_max_ = 0.14 e Å^−^^3^
203 parameters	Δρ_min_ = −0.16 e Å^−^^3^
1 restraint	Extinction correction: *SHELXL97* (Sheldrick, 2008), Fc^*^=kFc[1+0.001xFc^2^λ^3^/sin(2θ)]^-1/4^
Primary atom site location: structure-invariant direct methods	Extinction coefficient: 0.046 (5)

### Special details

**Table:**

Geometry. All s.u.'s (except the s.u. in the dihedral angle between two l.s. planes) are estimated using the full covariance matrix. The cell s.u.'s are taken into account individually in the estimation of s.u.'s in distances, angles and torsion angles; correlations between s.u.'s in cell parameters are only used when they are defined by crystal symmetry. An approximate (isotropic) treatment of cell s.u.'s is used for estimating s.u.'s involving l.s. planes.
Refinement. Refinement of *F*^2^ against ALL reflections. The weighted *R*-factor *wR* and goodness of fit *S* are based on *F*^2^, conventional *R*-factors *R* are based on *F*, with *F* set to zero for negative *F*^2^. The threshold expression of *F*^2^ > 2σ(*F*^2^) is used only for calculating *R*-factors(gt) *etc*. and is not relevant to the choice of reflections for refinement. *R*-factors based on *F*^2^ are statistically about twice as large as those based on *F*, and *R*- factors based on ALL data will be even larger.

### Fractional atomic coordinates and isotropic or equivalent isotropic displacement parameters (Å^2^)

**Table:**

	*x*	*y*	*z*	*U*_iso_*/*U*_eq_	
O1	0.4807 (4)	0.4173 (2)	0.83756 (18)	0.0698 (7)	
O2	0.7191 (4)	0.2587 (2)	0.86723 (18)	0.0693 (7)	
O3	0.6220 (4)	0.0581 (2)	0.7662 (2)	0.0713 (7)	
H3	0.6909	0.1050	0.8120	0.107*	
N1	0.0615 (5)	0.2188 (2)	0.18076 (18)	0.0540 (7)	
N2	0.2028 (4)	0.0436 (2)	0.03575 (18)	0.0485 (6)	
H2	0.3079	0.0004	0.0834	0.058*	
C1	0.5324 (6)	0.3081 (3)	0.8212 (2)	0.0499 (8)	
C2	0.3691 (5)	0.2334 (2)	0.7444 (2)	0.0421 (7)	
C3	0.4223 (5)	0.1106 (3)	0.7177 (2)	0.0492 (8)	
C4	0.2712 (6)	0.0446 (3)	0.6450 (2)	0.0577 (8)	
H4	0.3089	−0.0353	0.6286	0.069*	
C5	0.0600 (6)	0.0946 (3)	0.5943 (2)	0.0511 (8)	
C6	0.0011 (5)	0.2159 (3)	0.6212 (2)	0.0481 (8)	
C7	0.1630 (5)	0.2818 (2)	0.6959 (2)	0.0460 (7)	
H7	0.1275	0.3618	0.7128	0.055*	
C8	−0.1018 (7)	0.0297 (3)	0.5177 (3)	0.0691 (10)	
H8	−0.0694	−0.0504	0.4995	0.083*	
C9	−0.3021 (7)	0.0825 (4)	0.4708 (3)	0.0819 (12)	
H9	−0.4035	0.0387	0.4192	0.098*	
C10	−0.3613 (7)	0.2015 (4)	0.4978 (3)	0.0815 (12)	
H10	−0.5014	0.2359	0.4653	0.098*	
C11	−0.2116 (6)	0.2662 (3)	0.5721 (3)	0.0642 (9)	
H11	−0.2509	0.3450	0.5907	0.077*	
C12	0.2864 (6)	0.2257 (3)	0.1499 (3)	0.0621 (9)	
H12A	0.3974	0.1803	0.2017	0.074*	
H12B	0.3362	0.3107	0.1514	0.074*	
C13	0.2821 (6)	0.1748 (3)	0.0404 (3)	0.0582 (9)	
H13A	0.4357	0.1793	0.0224	0.070*	
H13B	0.1784	0.2230	−0.0123	0.070*	
C14	−0.0210 (5)	0.0334 (3)	0.0721 (2)	0.0559 (8)	
H14A	−0.0635	−0.0524	0.0751	0.067*	
H14B	−0.1396	0.0745	0.0208	0.067*	
C15	−0.0076 (6)	0.0901 (3)	0.1817 (2)	0.0572 (9)	
H15A	−0.1573	0.0843	0.2036	0.069*	
H15B	0.1034	0.0454	0.2339	0.069*	
C16	0.0568 (7)	0.2757 (3)	0.2852 (3)	0.0778 (11)	
H16A	0.1569	0.2313	0.3408	0.117*	
H16B	−0.0983	0.2742	0.2995	0.117*	
H16C	0.1086	0.3592	0.2841	0.117*	
C17	0.1976 (6)	−0.0108 (3)	−0.0721 (2)	0.0694 (10)	
H17A	0.1472	−0.0946	−0.0717	0.104*	
H17B	0.3498	−0.0079	−0.0898	0.104*	
H17C	0.0926	0.0347	−0.1249	0.104*	

### Atomic displacement parameters (Å^2^)

**Table:**

	*U*^11^	*U*^22^	*U*^33^	*U*^12^	*U*^13^	*U*^23^
O1	0.0622 (15)	0.0473 (14)	0.0920 (16)	0.0018 (12)	−0.0080 (11)	−0.0174 (12)
O2	0.0633 (17)	0.0680 (15)	0.0704 (14)	0.0035 (13)	−0.0053 (12)	−0.0016 (12)
O3	0.0711 (16)	0.0539 (14)	0.0857 (16)	0.0150 (13)	0.0058 (12)	0.0047 (12)
N1	0.0617 (18)	0.0417 (15)	0.0584 (15)	0.0002 (14)	0.0099 (12)	0.0000 (12)
N2	0.0493 (15)	0.0434 (14)	0.0510 (14)	0.0044 (12)	0.0042 (10)	0.0066 (12)
C1	0.049 (2)	0.047 (2)	0.0516 (18)	−0.0045 (16)	0.0016 (15)	0.0030 (15)
C2	0.0478 (18)	0.0365 (15)	0.0419 (15)	−0.0003 (14)	0.0074 (13)	0.0016 (13)
C3	0.055 (2)	0.0392 (17)	0.0534 (17)	0.0029 (15)	0.0085 (15)	0.0040 (15)
C4	0.079 (2)	0.0358 (16)	0.062 (2)	0.0007 (18)	0.0225 (17)	−0.0012 (15)
C5	0.066 (2)	0.0445 (19)	0.0440 (16)	−0.0126 (16)	0.0121 (15)	−0.0028 (14)
C6	0.0483 (19)	0.0502 (19)	0.0452 (15)	−0.0061 (15)	0.0069 (14)	0.0008 (15)
C7	0.057 (2)	0.0347 (15)	0.0462 (15)	0.0011 (15)	0.0089 (14)	−0.0039 (12)
C8	0.086 (3)	0.062 (2)	0.059 (2)	−0.021 (2)	0.0122 (19)	−0.0126 (18)
C9	0.078 (3)	0.101 (3)	0.061 (2)	−0.034 (3)	−0.0001 (19)	−0.008 (2)
C10	0.068 (3)	0.105 (3)	0.065 (2)	−0.015 (2)	−0.0048 (19)	0.013 (2)
C11	0.061 (2)	0.065 (2)	0.0631 (19)	0.0012 (19)	0.0015 (16)	0.0026 (17)
C12	0.065 (2)	0.0412 (18)	0.078 (2)	−0.0092 (17)	0.0058 (17)	0.0020 (17)
C13	0.063 (2)	0.0390 (17)	0.074 (2)	−0.0047 (16)	0.0165 (16)	0.0120 (15)
C14	0.0497 (19)	0.0503 (18)	0.067 (2)	−0.0031 (15)	0.0078 (15)	0.0032 (16)
C15	0.064 (2)	0.050 (2)	0.0585 (19)	−0.0035 (16)	0.0110 (16)	0.0051 (15)
C16	0.098 (3)	0.067 (2)	0.067 (2)	0.004 (2)	0.0124 (19)	−0.0089 (18)
C17	0.082 (3)	0.068 (2)	0.058 (2)	0.013 (2)	0.0097 (17)	0.0025 (18)

### Geometric parameters (Å, º)

**Table:**

O1—C1	1.253 (4)	C8—H8	0.9300
O2—C1	1.265 (4)	C9—C10	1.399 (6)
O3—C3	1.349 (4)	C9—H9	0.9300
O3—H3	0.8200	C10—C11	1.360 (5)
N1—C12	1.445 (4)	C10—H10	0.9300
N1—C16	1.455 (4)	C11—H11	0.9300
N1—C15	1.460 (4)	C12—C13	1.479 (5)
N2—C14	1.472 (4)	C12—H12A	0.9700
N2—C17	1.474 (4)	C12—H12B	0.9700
N2—C13	1.501 (4)	C13—H13A	0.9700
N2—H2	0.9100	C13—H13B	0.9700
C1—C2	1.479 (4)	C14—C15	1.497 (4)
C2—C7	1.361 (4)	C14—H14A	0.9700
C2—C3	1.427 (4)	C14—H14B	0.9700
C3—C4	1.360 (4)	C15—H15A	0.9700
C4—C5	1.400 (4)	C15—H15B	0.9700
C4—H4	0.9300	C16—H16A	0.9600
C5—C8	1.416 (4)	C16—H16B	0.9600
C5—C6	1.421 (4)	C16—H16C	0.9600
C6—C11	1.403 (4)	C17—H17A	0.9600
C6—C7	1.408 (4)	C17—H17B	0.9600
C7—H7	0.9300	C17—H17C	0.9600
C8—C9	1.348 (5)		
			
C3—O3—H3	109.5	C10—C11—C6	121.1 (4)
C12—N1—C16	112.7 (3)	C10—C11—H11	119.5
C12—N1—C15	108.7 (2)	C6—C11—H11	119.5
C16—N1—C15	110.6 (3)	N1—C12—C13	111.5 (3)
C14—N2—C17	112.5 (2)	N1—C12—H12A	109.3
C14—N2—C13	110.4 (2)	C13—C12—H12A	109.3
C17—N2—C13	111.9 (2)	N1—C12—H12B	109.3
C14—N2—H2	107.2	C13—C12—H12B	109.3
C17—N2—H2	107.2	H12A—C12—H12B	108.0
C13—N2—H2	107.2	C12—C13—N2	110.3 (3)
O1—C1—O2	123.0 (3)	C12—C13—H13A	109.6
O1—C1—C2	118.8 (3)	N2—C13—H13A	109.6
O2—C1—C2	118.2 (3)	C12—C13—H13B	109.6
C7—C2—C3	118.2 (3)	N2—C13—H13B	109.6
C7—C2—C1	120.1 (3)	H13A—C13—H13B	108.1
C3—C2—C1	121.7 (3)	N2—C14—C15	110.6 (2)
O3—C3—C4	119.4 (3)	N2—C14—H14A	109.5
O3—C3—C2	120.1 (3)	C15—C14—H14A	109.5
C4—C3—C2	120.6 (3)	N2—C14—H14B	109.5
C3—C4—C5	121.3 (3)	C15—C14—H14B	109.5
C3—C4—H4	119.4	H14A—C14—H14B	108.1
C5—C4—H4	119.4	N1—C15—C14	111.0 (2)
C4—C5—C8	123.2 (3)	N1—C15—H15A	109.4
C4—C5—C6	119.2 (3)	C14—C15—H15A	109.4
C8—C5—C6	117.6 (3)	N1—C15—H15B	109.4
C11—C6—C7	122.5 (3)	C14—C15—H15B	109.4
C11—C6—C5	119.6 (3)	H15A—C15—H15B	108.0
C7—C6—C5	117.9 (3)	N1—C16—H16A	109.5
C2—C7—C6	122.8 (3)	N1—C16—H16B	109.5
C2—C7—H7	118.6	H16A—C16—H16B	109.5
C6—C7—H7	118.6	N1—C16—H16C	109.5
C9—C8—C5	120.8 (4)	H16A—C16—H16C	109.5
C9—C8—H8	119.6	H16B—C16—H16C	109.5
C5—C8—H8	119.6	N2—C17—H17A	109.5
C8—C9—C10	121.7 (3)	N2—C17—H17B	109.5
C8—C9—H9	119.2	H17A—C17—H17B	109.5
C10—C9—H9	119.2	N2—C17—H17C	109.5
C11—C10—C9	119.2 (4)	H17A—C17—H17C	109.5
C11—C10—H10	120.4	H17B—C17—H17C	109.5
C9—C10—H10	120.4		
			
O1—C1—C2—C7	−2.5 (5)	C5—C6—C7—C2	−1.4 (4)
O2—C1—C2—C7	177.8 (3)	C4—C5—C8—C9	−179.6 (3)
O1—C1—C2—C3	176.7 (3)	C6—C5—C8—C9	1.2 (5)
O2—C1—C2—C3	−3.0 (4)	C5—C8—C9—C10	−1.8 (6)
C7—C2—C3—O3	−178.2 (3)	C8—C9—C10—C11	0.9 (6)
C1—C2—C3—O3	2.6 (4)	C9—C10—C11—C6	0.5 (6)
C7—C2—C3—C4	0.6 (4)	C7—C6—C11—C10	178.0 (3)
C1—C2—C3—C4	−178.6 (3)	C5—C6—C11—C10	−1.0 (5)
O3—C3—C4—C5	178.8 (3)	C16—N1—C12—C13	−176.1 (3)
C2—C3—C4—C5	−0.1 (4)	C15—N1—C12—C13	60.9 (3)
C3—C4—C5—C8	179.6 (3)	N1—C12—C13—N2	−58.3 (4)
C3—C4—C5—C6	−1.2 (4)	C14—N2—C13—C12	54.3 (3)
C4—C5—C6—C11	−179.0 (3)	C17—N2—C13—C12	−179.6 (3)
C8—C5—C6—C11	0.2 (4)	C17—N2—C14—C15	−179.8 (3)
C4—C5—C6—C7	1.9 (4)	C13—N2—C14—C15	−54.0 (3)
C8—C5—C6—C7	−178.9 (3)	C12—N1—C15—C14	−60.2 (3)
C3—C2—C7—C6	0.1 (4)	C16—N1—C15—C14	175.6 (3)
C1—C2—C7—C6	179.3 (3)	N2—C14—C15—N1	57.8 (3)
C11—C6—C7—C2	179.6 (3)		

### Hydrogen-bond geometry (Å, º)

**Table:**

*D*—H···*A*	*D*—H	H···*A*	*D*···*A*	*D*—H···*A*
O3—H3···O2	0.82	1.81	2.541 (3)	148
N2—H2···O1^i^	0.91	1.71	2.613 (3)	174

Symmetry code: (i) −*x*+1, *y*−1/2, −*z*+1.
